# Special Issue “The Fungal Cell Wall Integrity Pathway”

**DOI:** 10.3390/jof9030293

**Published:** 2023-02-24

**Authors:** Humberto Martín, María Molina

**Affiliations:** Departamento de Microbiología y Parasitología, Facultad de Farmacia, Instituto Ramón y Cajal de Investigaciones Sanitarias (IRYCIS), Universidad Complutense de Madrid, Plaza de Ramón y Cajal s/n, 28040 Madrid, Spain

Adaptation to external changes is necessary for all cell types to survive and thrive in diverse environments. Key to these responses are the MAPK-mediated signaling pathways, intracellular communication routes that sense external stimuli at the cell surface and are ubiquitous in the eukaryotic world [1]. Unlike mammalian cells, fungi are surrounded by a rigid structure, the cell wall, whose integrity must be preserved to prevent cell lysis. During the life cycle of fungi, the cell wall must be continuously modified without failing in robustness to allow cells to grow, divide or differentiate. Furthermore, in their natural habitats, the different fungal species are subjected to a wide variety of environmental stresses, including those challenging this outermost cell structure, which they must cope with using appropriate adaptive mechanisms [2]. First, studies in the early 1990s in the budding yeast *Saccharomyces cerevisiae* established that the cell wall integrity (CWI) pathway is the central MAPK cascade required both to monitor the structural and functional status of this essential cell envelope and to trigger an appropriate salvage response under conditions that perturb its integrity [3]. Subsequent studies in other fungal species, including plant and human pathogens, have provided evidence that this MAPK pathway is highly conserved in the fungal kingdom [4]. Therefore, understanding the CWI pathway-mediated compensatory mechanism is key for the development of efficient cell-wall-targeted antifungal therapies. Furthermore, recent efforts to delve deeper into this pathway have revealed that its functional role goes beyond the maintenance of this essential structure, reaching many other physiological aspects that have important implications in fungal growth and virulence [2].

In this Special Issue, expert researchers in this relevant subject have contributed with seven reviews and eleven original articles to advance our understanding of the CWI pathway by covering different structural, regulatory, and functional aspects in distinct yeasts and filamentous fungi. Yoshimi et al. summarize the current knowledge of CWI signaling in several filamentous fungal species, from sensor proteins required for the recognition of environmental changes to MAPK cascades and their targets involved in the regulation of cell wall polysaccharide synthesis genes [5]. To discuss the most important functional aspects, what is known in these fungi is compared to yeast species, such as the widely studied *Saccharomyces cerevisiae* or the pathogens *Candida* and *Cryptococcus*. In addition, they also address the role of the CWI pathway as a target for developing antifungal drugs and for manipulating the surface properties of fungi to improve productivity in industrial processes.

The importance of this signaling pathway in *Candida albicans* and its interconnections with other stress-activated pathways for remodeling the cell envelope under different stresses is reviewed by Ibe & Munro [6]. They analyze the key function of cell wall mannoproteins on the survival, growth, and virulence of this opportunistic pathogen, highlighting the value of understanding their molecular structure, activity, and regulatory mechanisms to identifying the most suitable diagnostic, therapeutic, and vaccine candidates, thus paving the way for better management of candidiasis. De Oliveira et al. focus their review on the CWI signaling in another yeast species, *Cryptococcus neoformans*, and its involvement in some of the virulence traits of this pathogen, such as thermotolerance, melanin synthesis, capsule growth, and Titan cell formation [7]. The ability of *C. neoformans* to enlarge cell size by increasing its protective external structure and undergoing such a characteristic morphological change has been extensively studied, as it allows the fungus to evade and resist the host immune system. These authors also emphasize the role of the CWI pathway as a pharmacological target that can be exploited to improve the effectiveness of existing antifungals as well as to develop new therapies.

The function of the CWI pathway beyond the regulation of cell wall homeostasis is discussed in the reviews on budding and fission yeast models provided by González-Rubio et al. and by Cansado et al., respectively. In the former, the authors summarize the currently available methodologies to identify phosphorylation targets of Slt2, the MAPK of the pathway, and compile all the genuine Slt2 substrates reported to date, whose variety of functions reflects the multiple processes regulated by the CWI pathway in *S. cerevisiae* [8]. They also provide a list of putative Slt2 substrates, ready to be confirmed in future works. The second review also positions the CWI as a multi-faceted pathway that impacts multiple functional aspects of the *Schizosaccharomyces pombe* life cycle both during growth and in response to stress [9]. Among the processes described as regulated by the CWI signaling in these yeasts, besides the maintenance of cell wall integrity, are gene transcription, control of mRNA stability or transport through RNA-binding proteins, regulation of calcium homeostasis, interplay with PKA and TOR pathways, control of cell cycle, and modulation of cytokinesis. These last two aspects are the topics reviewed in detail, respectively, by Quilis et al. and Roncero et al. The CWI pathway is presented by the former authors as a versatile toolbox to arrest cell cycle progression in budding yeast exposed to unfavorable conditions, due to its role in regulating major cell cycle transitions in response to cell surface perturbance or genotoxic stress [10]. They describe the mechanisms by which the CWI pathway impinges on different cell cycle regulators and checkpoints to delay cell cycle progression until the damage is repaired, in order to resume cell division with all the guarantees for cell survival. Roncero et al. first summarize the key steps of yeast cytokinesis, including ring assembly, septum formation, and cell separation, to subsequently analyze the multiple interconnections of the CWI signaling responses with these processes in both *S pombe* and *S. cerevisiae*, highlighting the differences between these two model yeasts with distinct modes of growth [11].

The research papers in this Special Issue present very significant insights into different aspects of various key components of this signaling pathway, ranging from receptors to effectors. Starting with the former, Schöppner et al. address the relationship between the structure and functional properties of the ScWsc1 receptor [12]. By solving a high-resolution crystal structure of the extracellular cysteine-rich domain (CRD) of yeast Wsc1, they show that the protein surface of the CRD contains three aromatic clusters, which play an essential role under cell wall stress conditions. Conservation of these functional hotspots among other fungal Wsc sensors enhances their relevance. In a complementary study, Voskoboynikova et al. succeeded in extracting this mechanosensor from the yeast plasma membrane using a detergent-free procedure into a semi-native lipid environment [13]. The use of the amphipathic styrene-maleic acid (SMA) and SMA-related copolymers allowed for the formation of SMALPS (SMA/lipid particles) that accommodate only single sensor molecules. Analysis of the preparations by dynamic light scattering (DLS), fluorescence correlation spectroscopy (FCS), and single-particle transmission electron microscopy (TEM) allowed for the deduction of the first three-dimensional structural model of Wsc1. This work confirms the tripartite organization of Wsc1, provides novel clues on the structure–function relationship of this sensor, and shows the method for studying other membrane proteins. From a functional perspective, an unresolved issue is the role of the distinct receptors that feed the CWI pathway. The work of Hall et al. provides additional information on this aspect: whereas Mid2 is necessary during shmoo formation, Wsc1 is of critical importance in zygote and diploid survival [14]. Interestingly, activation of Pkc1 can compensate for the absence of the receptor, suggesting that its CWI signaling role is essential for this functionality. Other functions of CWI receptors are described by Montella-Manuel et al., who demonstrate that Mtl1 plays a critical role in sensing reductions in glucose concentration, in order to trigger autophagy during diauxic transition [15]. In addition, it is also involved in the autophagic degradation of mitochondria during the stationary phase. Here, activation of CWI components does not restore the lack of bulk autophagy activation observed in *mtl1* mutants, suggesting a CWI-independent function for this sensor. Unraveling the role of the different receptors of the CWI pathway in cellular physiology promises to be an interesting field of research in the coming years.

This issue also provides new data that refine our understanding of the complex regulation and functional roles of Pkc1, the top kinase of the CWI pathway. Liu et al. demonstrate that this kinase is hyperphosphorylated in response to DNA damage in a Hrr25-dependent manner. Hrr25 (HO and Radiation Repair) is an orthologue of casein kinase 1 (CK1) and is involved in the transcriptional response to DNA damage [16]. Mutation of CK1 consensus sites in Pkc1 prevents Pkc1 hyperphosphorylation and reduces transcriptional induction by genotoxic stress, suggesting that hyperphosphorylation of Pkc1 by Hrr25 contributes to this response. In turn, Sellers-Moya et al. prove that, in contrast to other CWI stimuli, Pkc1 is not essential for the activation of the CWI MAPK module in response to clotrimazole [17]. This imidazole activates both the HOG and CWI pathways and promotes the appearance of a low-mobility form of Slt2 caused by additional phosphorylation to that occurring in the conserved TEY activation motif. The oxidative stress generated by this antifungal drug is linked to Tpk3-mediated PKA activity and is responsible for all these effects. The function of the CWI pathway and in particular the MAPK Slt2 are also expanded in the work of Sánchez-Adriá et al., who show the role of Slt2 in downregulating TORC1 signaling in response to ER (Endoplasmic Reticulum) stress [18]. In this situation, the CWI pathway is involved in the activation of autophagy and the increased synthesis of ATP. Furthermore, these authors show that Slt2 is required for *GFA1* expression, coding the glutamine:fructose-6-phosphate amidotransferase, a key enzyme within the conserved hexosamine biosynthetic pathway (HBP) essential for different metabolic pathways. Taken together, these results suggest the importance of Slt2 in modulating the bioenergetics of the protective response under ER stress, as well as confirm the key role of the CWI pathway in the crosstalk between different signaling pathways.

Novel components participating in the maintenance of cell wall integrity are also uncovered. By performing two high-throughput screenings using the yTHC collection of yeast conditional mutant strains, Sanz et al. provide insights into essential genes required to cope with cell wall damage conditions, as well as those affecting CWI-associated gene expression [19]. This work reveals how the RSC chromatin remodeling complex, unveiled in both screenings, is likely to cooperate with SWI/SNF and SAGA complexes for the chromatin remodeling necessary for the transcriptional activation of CWI-dependent genes upon stress. The paper by Ghanegolmohammadi et al. follows a distinct and novel approach in order to increase our understanding of the function of cell wall mannoproteins [20]. To this end, they investigate 32 mannoprotein mutants in *S. cerevisiae* using high-dimensional morphological phenotyping. Comprehensive analysis of their morphological phenotypes provides strong clues about the functions of the affected proteins belonging to the same cluster of mutants, in particular the mechanistic and functional roles of the distinct glycoproteins in cell morphogenesis.

Finally, two contributions reflect how evolution, using conserved elements, has shaped signaling pathways in fungi. In this line, the work of Navarro et al. shows that the dimorphism-regulating histidine kinase (Drk1) of the pathogenic fungus *Paracoccidioides brasiliensis* plays an important role in cell wall homeostasis, in contrast to the involvement of histidine kinases in the response to high osmolarity in *S. cerevisiae* [21]. In turn, Gomez-Gil et al. investigate the architecture and functionality of the CWI pathway (CIP pathway) of *Schizosaccharomyces japonicus* [22]. They prove that, while certain features of this pathway previously described in *S. pombe* are conserved in *S. japonicus*, others have evolved differently in both fission yeast species.

In sum, this Special Issue covers many, although not all, aspects of the signaling mechanisms and functions of the CWI pathway. It is our sincere hope that the articles presented here serve general readers by providing the most up-to-date information on this important aspect of fungal biology, as well as inspiring new ideas and challenging questions for researchers in the coming years.

## Data Availability

Not applicable.
